# Model-Based Comparison of Deep Brain Stimulation Array Functionality with Varying Number of Radial Electrodes and Machine Learning Feature Sets

**DOI:** 10.3389/fncom.2016.00058

**Published:** 2016-06-10

**Authors:** Benjamin A. Teplitzky, Laura M. Zitella, YiZi Xiao, Matthew D. Johnson

**Affiliations:** ^1^Department of Biomedical Engineering, University of MinnesotaMinneapolis, MN, USA; ^2^Institute for Translational Neuroscience, University of MinnesotaMinneapolis, MN, USA

**Keywords:** Deep brain stimulation, DBS, computational modeling, machine learning, neuromodulation, DBS lead, DBS programing algorithms

## Abstract

Deep brain stimulation (DBS) leads with radially distributed electrodes have potential to improve clinical outcomes through more selective targeting of pathways and networks within the brain. However, increasing the number of electrodes on clinical DBS leads by replacing conventional cylindrical shell electrodes with radially distributed electrodes raises practical design and stimulation programming challenges. We used computational modeling to investigate: (1) how the number of radial electrodes impact the ability to steer, shift, and sculpt a region of neural activation (RoA), and (2) which RoA features are best used in combination with machine learning classifiers to predict programming settings to target a particular area near the lead. Stimulation configurations were modeled using 27 lead designs with one to nine radially distributed electrodes. The computational modeling framework consisted of a three-dimensional finite element tissue conductance model in combination with a multi-compartment biophysical axon model. For each lead design, two-dimensional threshold-dependent RoAs were calculated from the computational modeling results. The models showed more radial electrodes enabled finer resolution RoA steering; however, stimulation amplitude, and therefore spatial extent of the RoA, was limited by charge injection and charge storage capacity constraints due to the small electrode surface area for leads with more than four radially distributed electrodes. RoA shifting resolution was improved by the addition of radial electrodes when using uniform multi-cathode stimulation, but non-uniform multi-cathode stimulation produced equivalent or better resolution shifting without increasing the number of radial electrodes. Robust machine learning classification of 15 monopolar stimulation configurations was achieved using as few as three geometric features describing a RoA. The results of this study indicate that, for a clinical-scale DBS lead, more than four radial electrodes minimally improved in the ability to steer, shift, and sculpt axonal activation around a DBS lead and a simple feature set consisting of the RoA center of mass and orientation enabled robust machine learning classification. These results provide important design constraints for future development of high-density DBS arrays.

## Introduction

Deep brain stimulation (DBS) is a neurosurgical intervention for symptomatic treatment of a number of brain disorders. The success of DBS therapy relies on accurate electrode placement within the brain (Rezai et al., 2006) and generation of spatially defined tissue voltage distributions that can precisely modulate brain activity with millimeter, or even sub-millimeter resolution (Butson et al., 2007). The size of the anatomical targets, and their proximity to neural pathways that when stimulated generate unwanted side effects, make selective modulation challenging for this therapy. Commercial DBS leads currently consist of a stack of cylindrical shell electrodes that can accommodate current steering along the lead axis (Wei and Grill, 2005; Chaturvedi et al., 2012; Barbe et al., 2014a,b). Such current steering can be useful for enhancing the ability to target the subthalamic nucleus (Kuncel and Grill, 2004; Butson and McIntyre, 2008; Frankemolle et al., 2010), globus pallidus (Johnson and McIntyre, 2008; Johnson et al., 2012), and motor thalamus (Kuncel and Grill, 2004; Butson and McIntyre, 2008; Keane et al., 2012). However, the cylindrical electrode design of current DBS leads produces predominantly axisymmetric modulation of neuronal activity (Keane et al., 2012). This axisymmetric modulation enables inadequate flexibility to adapt stimulation to compensate for neurosurgical targeting errors tangential to the DBS lead (Martens et al., 2011; Keane et al., 2012) or for targeting anatomical regions with complex geometries (Zitella et al., 2013; Teplitzky et al., 2014). In such cases, delivering therapy without evoking side effects such as phantom sensory perceptions, involuntary motor contractions, and cognitive/mood changes can be challenging (Frankemolle et al., 2010; Chaturvedi et al., 2012; Keane et al., 2012).

The concept of current steering with implantable electrode arrays has existed in the fields of spinal cord stimulation (Holsheimer et al., 1998; Manola et al., 2007), intracochlear stimulation (Firszt et al., 2007; Berenstein et al., 2008), and retinal stimulation (Matteucci et al., 2013; Dumm et al., 2014) for some time. Recent computational and experimental work has also applied this concept to preclinical and clinical DBS electrode arrays, which employ three to four radially distributed electrodes per row and several rows per lead (Buhlmann et al., 2011; Martens et al., 2011; Contarino et al., 2014; Cubo et al., 2014; Pollo et al., 2014; Bour et al., 2015). Such DBS arrays (DBSAs) have potential to improve steering, shifting, and sculpting of neural activation beyond the capacity of conventional DBS leads with cylindrical shell electrodes. However, it is presently not clear how the number of radial DBSA electrodes impact the ability to steer, shift, and sculpt a region of neural activation (RoA).

In addition to the challenges associated with understanding current steering with DBS arrays, leads with more than the conventional four electrodes have the potential to create significant patient programming challenges. Currently, clinicians select programing settings for a patient using trial-and-error through a monopolar review. A clinician will systematically stimulate through each of the available electrodes using increasing stimulation amplitudes, evaluate the patient's symptoms and the presence of side effects, and select the optimal stimulation configuration for the patient (Volkmann et al., 2006). With only four electrodes this can be a time consuming and imprecise task. Increasing the number of electrodes has the potential to greatly complicate this problem, making programming impractical or even infeasible in a clinical setting. To address this issue, model based optimization algorithms (Xiao et al., 2016) and machine learning classifiers (Chaturvedi et al., 2013) have been proposed. In general, the goal of these algorithms is to use medical imaging to determine the location of an implanted DBS lead relative to the targeted brain region and using this information, predict potentially therapeutic stimulation settings in order to guide the clinician in programming the implanted DBS system. Implementation of such techniques; however, relies heavily on the identification of robust quantifiable measures, or features, that describe the desired region or volume of activation. Currently, it remains unclear which RoA features are best used in combination with machine learning classifiers to predict programming settings to target a particular area near a DBSA.

In the first section of this manuscript we used computational modeling to explore DBSA lead design and current steering strategies. In particular, we calculated the maximum stimulation amplitude for various DBSA designs in the context of charge injection and charge storage capacity limits. We then investigated the size, shape, and location of a region of neural activation resulting from stimulation using a variety of electrode configurations within these limits. In the second section of this manuscript, we evaluate various machine learning feature sets for predicting stimulation settings to target a particular region near the DBS lead.

## Materials and methods

### Radially segmented DBS arrays

**Figure 1:**
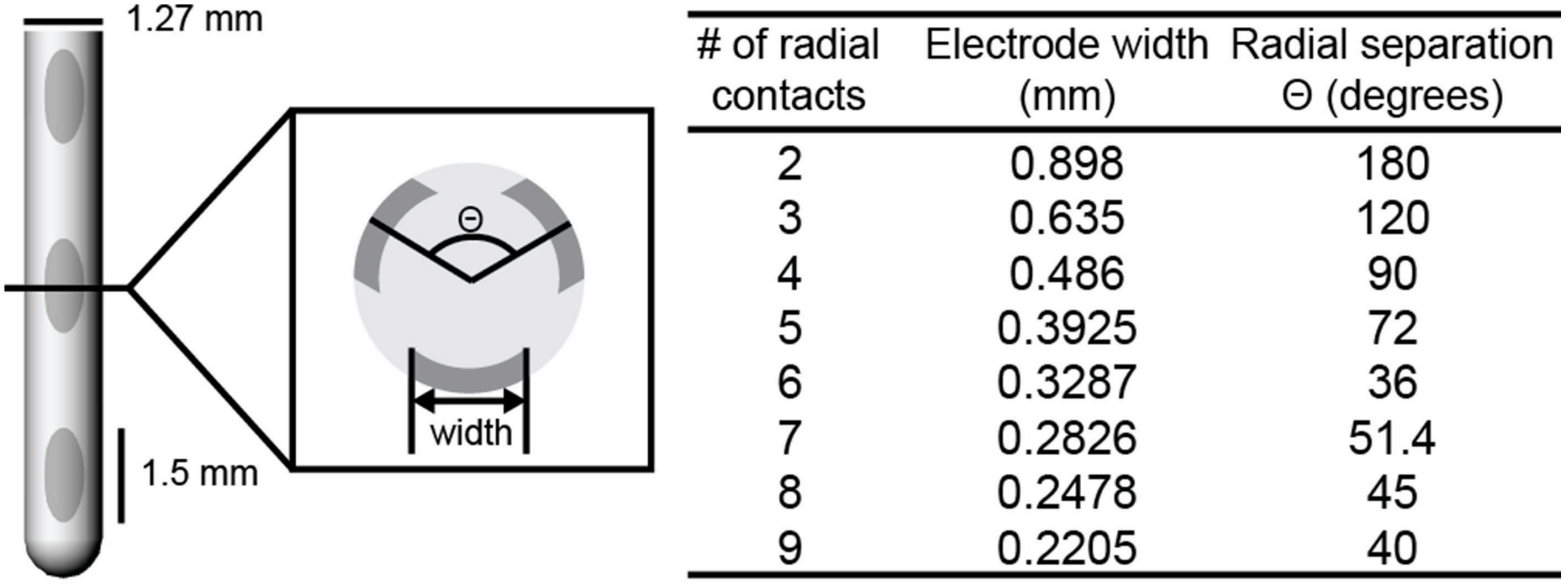
**DBSA lead design**. DBSA leads were designed with two to nine electrodes per row. DBSA electrode width and radial separation were calculated for each lead design using Equations (1, 2). The DBSA–e3–h1.5 lead design is shown. Electrode height was 1.5 (shown), 1.0, or 0.5 mm.

Twenty-four deep brain stimulation array (DBSA) and three non-array leads were created in COMSOL Multiphysics v4.4. DBSA leads included two to nine electrodes per row. Each DBSA electrode was constructed by projecting an ellipse onto the cylindrical lead body and extruding the resulting surface 0.1 mm into the lead body. The width of the projected ellipse (Figure 1) was calculated using the equation of a chord whose endpoints lie on a circle with a diameter equal to the lead body diameter, 1.27 mm (Equations 1, 2).

(1) $$\begin{array}{rcl} \Theta & = & \frac{360}{n} \end{array}$$

(2) $$\begin{array}{rcl} electrode\;width & = & d*\text{sin}\left(\frac{\Theta }{2}\right) \end{array}$$

where Θ was the center-to-center electrode separation, *d* was the lead body diameter, and *n* was the number of radial electrodes in a row. Non-array leads included conventional cylindrical shell electrodes. Both array and non-array electrodes were constructed with three heights: 0.5, 1.0, and 1.5 mm. Each DBS lead included four rows of electrodes and the separation between rows was equal to electrode height. Each lead diameter was 1.27 mm in accordance with the diameter of the clinical Medtronic 3387 and 3389 DBS leads (Medtronic Inc., Minneapolis, MN). To simplify reference to each DBS lead design, the following naming convention was implemented: DBSA–e[number of radial electrodes]–h[electrode height]. For example, DBSA–e4–h1.5 would refer to the DBSA lead with 4 radial electrodes per row, each with a height of 1.5 mm.

### Tissue conductance models

**Figure 2:**
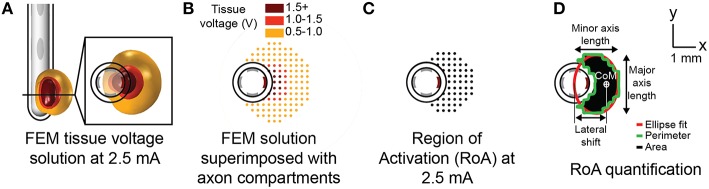
**Modeling axonal activation**. Tissue voltage during stimulation was modeled for each stimulation configuration using the finite element method **(A)**. The multi-compartment axon model population superimposed with extracellular potentials derived from the tissue voltage predictions **(B)**. A spatial axonal activation profile, or region of activation (RoA) plot resulting from stimulation at 2.5 mA **(C)**. RoA quantification using regional properties calculated from a closed binary image of the RoA plot **(D)**.

Simulations were conducted using only the bottom row of electrodes for each lead. A three-dimensional tissue conductance model was created for each stimulation configuration using COMSOL Multiphysics v4.4 and solved for using the finite element method (FEM; Figure 2A). Each tissue model incorporated a lead body (σ = 1e−12 S/m), electrodes (σ = 1e6 S/m), a 0.25 mm thick encapsulation layer (σ = 0.18 S/m; Grill and Mortimer, 1994; Lempka et al., 2009), and a 20 cm diameter sphere representing bulk neural tissue (σ = 0.3 S/m; (Ranck, 1963; Stances, 1975). Point current-sources were placed at the three-dimensional center of each electrode. The surface of the bulk neural tissue sphere was set to ground, i.e., zero volts, via Dirichlet boundary conditions. A variable resolution mesh containing quadratic tetrahedral elements ranging from 0.2 mm near the electrode to 10 mm near the model perimeter was generated via Delaunay triangulation. The resulting mesh contained 280,000–310,000 elements depending on the lead design. To confirm that further mesh refinement was not advantageous, the average relative change in the calculated potentials were determined at the midpoint of each axon model compartment using a mesh with elements that were two and three times smaller than the previously described model. The average relative change in the calculated potentials was found to be < 1% for these more refined models.

To investigate impact of the changes to the electrode-tissue interface (ETI) resulting from novel electrode geometries, a three-element Randles equivalent circuit model of the ETI was constructed for the lead with the smallest and largest electrode surface areas. In these models, the Fourier FEM described by Butson and McIntyre (2005) was implemented so that capacitive effects of the ETI could be captured. Briefly, the Fourier FEM was carried out by creating a waveform with a 90 μs cathodic pulse in the time domain (*dt* = 1 μs), performing the 1024 point discrete Fourier transform (DFT), solving the finite element model (tissue $\varepsilon_{\text{r}}=1\times 10^{6}$, Gabriel S. et al., 1996) at each of the 513 frequencies represented within the DFT (0–512 kHz), scaling and phase shifting the finite element model results by the DFT magnitude and phase, and finally performing the 1024 point inverse DFT on the result to reconstruct the stimulation waveform in the time domain. The equivalent circuit model was represented at the electrode surface within the frequency dependent finite element model as a circuit terminal using the COMSOL Multiphysics AC/DC module. In accordance with previous work (Howell et al., 2014), the equivalent circuit model included an access resistance, R_a_, in series with a parallel RC pair consisting of a faradaic resistance, R_f_, and double layer capacitance, C_dl_. R_a_ was calculated using the finite element model solution for 1 v applied at the electrode surface from which the effective applied current was calculated by integrating the normal current density across the electrode surface and taking the reciprocal. R_f_ and C_dl_ were calculated from the distributed faradaic resistance (150 Ω-cm^2^, Wei and Grill, 2009) and the distributed double layer capacitance (30 μF/cm^2^, Wei and Grill, 2009) using the electrode surface area. Inclusion of the ETI was confirmed to have no discernable impact on the stimulation results, and thus the ETI equivalent circuit model was excluded from subsequent simulations.

### Stimulation configurations

Current-regulated stimulation was modeled using one or multiple independent sources. Variations on stimulation configuration were constrained to monopolar settings and included single-cathode stimulation, uniform multi-cathode stimulation, and non-uniform multi-cathode stimulation. Uniform multi-cathode stimulation involved uniformly splitting the total cathodic current across all designated cathodes. Non-uniform multi-cathode stimulation involved assigning different proportions of total cathodic current to a single, primary cathode, and evenly distributing the remaining cathodic current across the remaining electrodes in a given row. Simulations of 15 monopolar single-cathode and uniform multi-cathode stimulation configurations using only the DBSA–e4–h1.5 lead were used for machine learning feature set analysis.

### Multi-compartment axon models

Three-dimensional multi-compartment myelinated axon models were distributed within a lead-centered 13-by-13 mm grid. Axons were separated by 0.25 mm and aligned parallel to the DBS lead. While the axon model orientations were generated in an artificial framework, the orientations were generally similar to fiber tracts (e.g., corticospinal tract of internal capsule) (Chaturvedi et al., 2013) that course approximately parallel to clinical DBS lead targets (e.g., subthalamic nucleus DBS) and that are hypothesized to elicit side effects when stimulated (Tommasi et al., 2008). Fibers were modeled with a 2 μm diameter (Kamiya et al., 2014) and populated with compartments representing nodes of Ranvier, myelin attachment segments, paranode main segments, and internode segments connected through an axial resistance. Axon compartment properties were consistent with the multi-compartment cable model axon developed and described in detail by McIntyre et al. (2004).

Rather than incorporating tissue conductance using the computationally expensive Fourier FEM method, the quasistatic solution at each axon compartment was scaled by a time-varying experimentally-recorded 135 Hz charge-balanced current-regulated stimulation waveform (Lempka et al., 2010; Equation 3).

(3) $$\begin{array}{l} \Phi \left(x,y,z,t\right)=\Phi \left(x,y,z\right)*w\left(t\right) \end{array}$$

Extracellular potential, represented by Φ for a given model axon compartment was scaled by the time varying 135 Hz waveform, *w(t)*. The charge-balanced waveform consisted of a 90 μs pulse followed by a 400 μs interphase delay and a 3 ms pulse with opposite polarity. The waveform-scaled extracellular potential was dynamically incorporated into the model axon compartments (Figure 2B) using the Neuron programming environment v7.3 (Hines and Carnevale, 1997). Within the Neuron programming environment, the axonal membranes were perturbed by driving membrane current using the *extracellular* mechanism (*e_extracellular)*, with parameters consistent with previous work (Teplitzky et al., 2014).

### Calculating neural activation thresholds and regions of activation

The total applied cathodic current threshold for inducing axonal spiking was calculated for each model axon within each tissue voltage model using a binary threshold-searching algorithm. The algorithm relied upon trial-and-error within a narrowing range of stimulation amplitudes that was considered to have converged once the range of stimulation amplitudes was reduced to 0.01 mA. Axons were considered “activated” if an action potential was recorded within 3 ms of stimulation following 8 out of 10 stimulation pulses at the distal node of Ranvier. For each stimulation configuration, two-dimensional spatial activation plots, referred to as region of activation (RoA) plots, were generated by plotting the cross-section of the axon population with activation-thresholds less than or equal to a specified stimulation amplitude (Figure 2C). Where charge storage capacity and charge injection limits were considered, the maximum safe stimulation amplitude was calculated using Equations (4, 5), respectively. The reversible charge storage capacity, 150 μC/cm^2^, represented the upper limit of reported values (Merrill et al., 2005; Cogan, 2008) for platinum-iridium electrodes like those generally used in DBS for cathodic-pulse leading charge balanced waveforms. The charge injection limit was characterized by a safety factor, *k* = 2.0, was derived from the charge per phase verses charge density per phase relationship (McCreery et al., 1990; Merrill et al., 2005) as a limit for safe charge delivery to neural tissue.

(4) $$\begin{array}{rcl} I_{CSC} & = & \frac{CSC\;\times A}{pw} \end{array}$$

(5) $$\begin{array}{rcl} I_{SF} & = & \frac{\sqrt{A\;\times 10^{k}}}{pw} \end{array}$$

With stimulation amplitude in amperes, *I*; charge storage capacity in μC/cm^2^, *CSC*; surface area of a single electrode in cm^2^, *A*; and cathodic pulse-width, *pw*.

### RoA quantification

Binary image analysis techniques were used to extract quantifiable metrics from each RoA at amplitudes ranging from 1 to 5 mA in 0.1 mA increments resulting in 41 RoAs per stimulation configuration. These techniques were used for quantification rather than precise measurement of the spatial activation profile to ensure that the process could be replicated in the context of post-operative medical imaging for the purpose of patient programing. Post-processing began with saving RoA plots spanning the 13-by-13 mm axon-space within 20-by-20 cm lead-centered images. A binary transform of each image was performed and morphologically closed using disk-shaped elements in order to preserve the ellipsoidal nature of the region. Regional properties including area, perimeter, center-of-mass (CoM), major axis length, and minor axis length were extracted from each of the closed images (Figure 2).

From these regional properties, several metrics were calculated to compare lead designs. These included lateral shift, angular shift, aspect ratio, target region coverage, and target region overspill. Lateral shift was calculated as distance from the lead-center to the RoA CoM in the direction of the primary cathode (usually along the *x*-axis). Angular shift, in the context of single-cathode stimulation through two neighboring electrodes, was calculated as the angle, in degrees, between vectors running from the lead-center to each RoA CoM. Aspect ratio was calculated as the RoA minor axis length divided by the RoA major axis length. Target region coverage and overspill were calculated for a set of experiments where a target region was placed between neighboring electrodes. These experiments were run using only the DBSA–e4–h1.5, which has electrodes separated by 90°. The target region, therefore, was generated from the same lead but was rotated 45° about the lead-center. Overlap between the target region and the activated region was calculated by first multiplying the binary image transforms of the two regions and then calculating the percent of the target region area covered by the overlapped region. Overspill was estimated by multiplying the binary image transforms of the activated region and the inverse of the target region, and then calculating the resulting area in mm^2^. Overlap and overspill were calculated for stimulation amplitudes ranging from 1 to 5 mA at 0.1 mA increments using three monopolar configurations. All processing and calculations of regional properties were performed using the Matlab Image Processing Toolbox (v2014b).

### Feature sets

**Figure 3:**
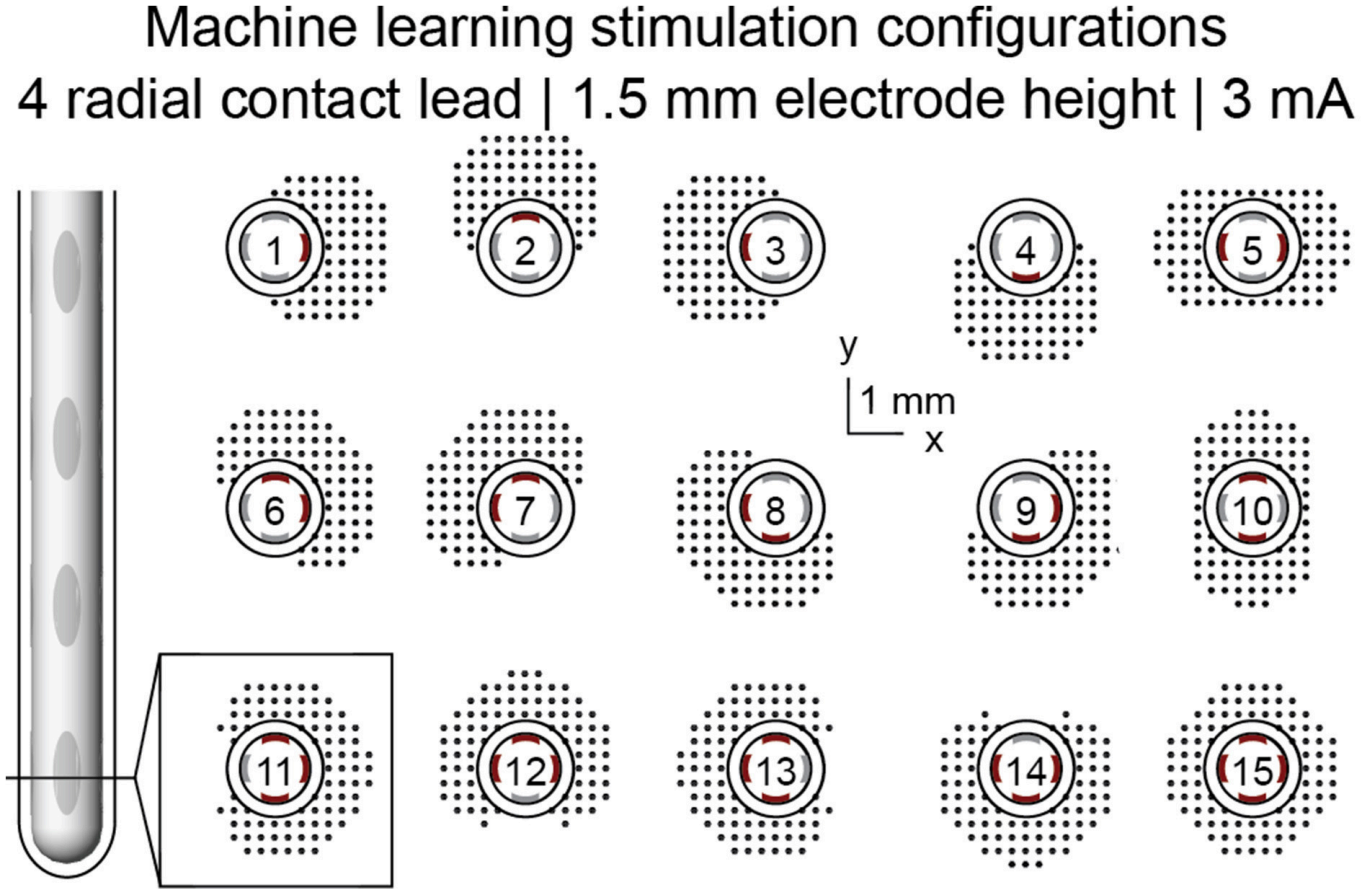
**Machine learning feature set generation**. Machine learning features were extracted from simulation results spanning 15 monopolar stimulation configurations at simulation amplitudes ranging from 1 to 5 mA in 0.1 mA increments.

Feature sets (Table 1) were derived from simulations of 15 monopolar stimulation configurations using the DBSA–e4–h1.5 lead (Figure 3). Because RoA measures were conducted at 41 amplitudes (1 to 5 mA in 0.1 mA increments) using 15 stimulation configurations, feature sets for 41 × 15 = 615 RoAs were generated. Post-processing of RoA plots was performed using the same binary image analysis techniques as described in Section RoA Quantification. From the post-processed binary images, three feature sets were generated: a region properties feature set (RPFS), a Legendre polynomial feature set (LPFS; Giselsson et al., 2013), and a 7 Hu invariant moments feature set (7 HuIM; Hu, 1962). The RPFS included the common region properties; center or mass, area, perimeter, convex hull area, solidity as well as features derived from an ellipse fit to the RoA; eccentricity, orientation, major axis length, and minor axis length. The LPFS was generated using the distance transform of each RoA binary image. The distance transform results were sorted in ascending order, normalized to the largest value, and fit to a 9th order Legendre polynomial. The features consisted of the coefficients of this 9th order Legendre polynomial. The majority of features that were investigated originate from computer vision applications where desirable traits include invariance to scale, rotation, and translation (Lowe, 1999). We hypothesized that the ideal feature set for prediction of stimulation configuration would (1) be rotation and translation variant since RoA direction underlies current steering, and (2) scale invariant with regard to stimulation amplitude but not with regard to RoA offset. To achieve this, distance of the RoA CoM from lead-center in the x and y directions were included in each feature set.

**Table 1:** **Features extracted from 15 monopolar stimulation configurations using the DBSA–e4–h1.5 lead**.

**Number**	**Feature**
1	Center of mass *x*-coordinate
2	Center of mass *y*-coordinate
3	Eccentricity of ellipse fit
4	Orientation of ellipse fit
5	Major axis length of ellipse fit
6	Minor axis length of ellipse fit
7	Area
8	Perimeter
9	Convex hull area
10	Solidity
11–20	Legendre polynomial coefficients from distance transform (Giselsson et al., 2013)
20–27	7 Hu invariant moments (Hu, 1962)

*Twenty-seven features were extracted from each RoA. The region properties feature set (RPFS) included features 1 through 10, the Legendre polynomial feature set (LPFS) included features 1, 2, and 11 through 20, and the 7-Hu invariant moments feature set (7 HuIM) included features 1, 2, and 20 through 27*.

### Classification and feature set quality assessment

Each of the 615 samples in the proposed classification problem included all features (Table 1) from a single RoA. The goal of the classification problem was to classify each sample, using a subset of features i.e., one of the three feature sets, as originating from the correct stimulation configuration, of which there were 15. The quality of each feature set was assessed using 10-fold cross validation of five classification models: k-nearest neighbor (KNN), naïve Bayes (NB), a multi-class support vector machine (mSVM) with a radial-basis function kernel (Lauer and Guermeur, 2011), a two-layer feed-forward pattern recognition neural network (NN) with 20 hidden elements, and a random forest (RF) decision tree ensemble with 100 trees (Breiman, 2001). All models except the mSVM were implemented using the Matlab Statistics Toolbox (v2014b). Training and testing data sets were pseudo-randomly divided within each cross validation fold such that each class was represented approximately equally and no samples were used for both training and testing. Classification accuracy was calculated for each fold as the number of correctly classified samples divided by the number of classified samples. The mean accuracy and standard error of the accuracy were then calculated across all 10 folds.

Feature importance was assessed using sequential forward selection and Breiman's random forest algorithm. Sequential forward selection was performed using the neural network and naïve Bayes classifiers. In each case, starting with an empty feature set, the classifier was run using each of the 27 features and the feature with the highest accuracy was considered the most important and added to the feature set. Classification was then performed using each of the remaining 26 features in combination with the first elected feature, and again the feature with the highest accuracy was considered the most important and added to the feature set. This process was repeated until the feature set contained 10 of the 27 features. From the random forest classifier, feature importance was assessed by calculating the increase in prediction error that resulted from random permutation of each feature across the out-of-bag samples. Features with the greatest effect on error were considered the most important.

## Results

### Stimulation amplitude limits

**Figure 4:**
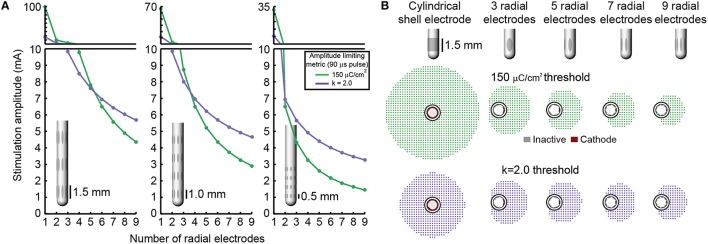
**Stimulation amplitude limits**. Maximum stimulation amplitude (for a biphasic waveform with a 90 μs initial pulse) was calculated for each lead design using a charge storage capacity of 150 μC/cm^2^ and a safety factor limit of *k* = 2.0 **(A)**. RoAs resulting from stimulation amplitude limits for several example DBSA lead designs **(B)**.

Increasing the number of radial electrodes resulted in a reduced electrode surface area. This in-turn lowered the theoretical stimulation amplitude that could be safely delivered through each electrode to neural tissue. More precisely, as the number of radial electrodes was increased both charge storage capacity and charge injection constraints limited the safe stimulation amplitude. This relationship followed an exponentially decaying trend (Figure 4). Charge injection constraints limited stimulation amplitude for leads with five or fewer radial electrodes with an electrode height of 1.5 mm. Charge storage capacity limited the stimulation amplitude for leads with more than five radial electrodes and electrode height of 1.5 mm. As electrode height was decreased, the intersection of the two lines: charge storage capacity constrained amplitude and charge injection constrained amplitude was shifted left, toward a smaller number of radial electrodes. Charge storage capacity was found to be the limiting factor for all DBSAs with an electrode height of 0.5 mm. In accordance with the inclusion of surface area in Equations (4,5), stimulation amplitude limited by charge storage capacity was proportional to the electrode height, while stimulation amplitude limited by charge injection was proportional to the square root of electrode height. Most electrode designs (23/27) were limited to stimulation amplitudes below 10 mA per electrode, while approximately half (13/27) were limited to amplitudes below 5 mA using the 150 μC/cm^2^ and *k* = 2.0 limits. All DBSA designs with an electrode height of 0.5 mm were limited to amplitudes below 5 mA per electrode.

### Steering, shifting, and sculpting activation with single-cathode monopolar DBS

**Figure 5:**
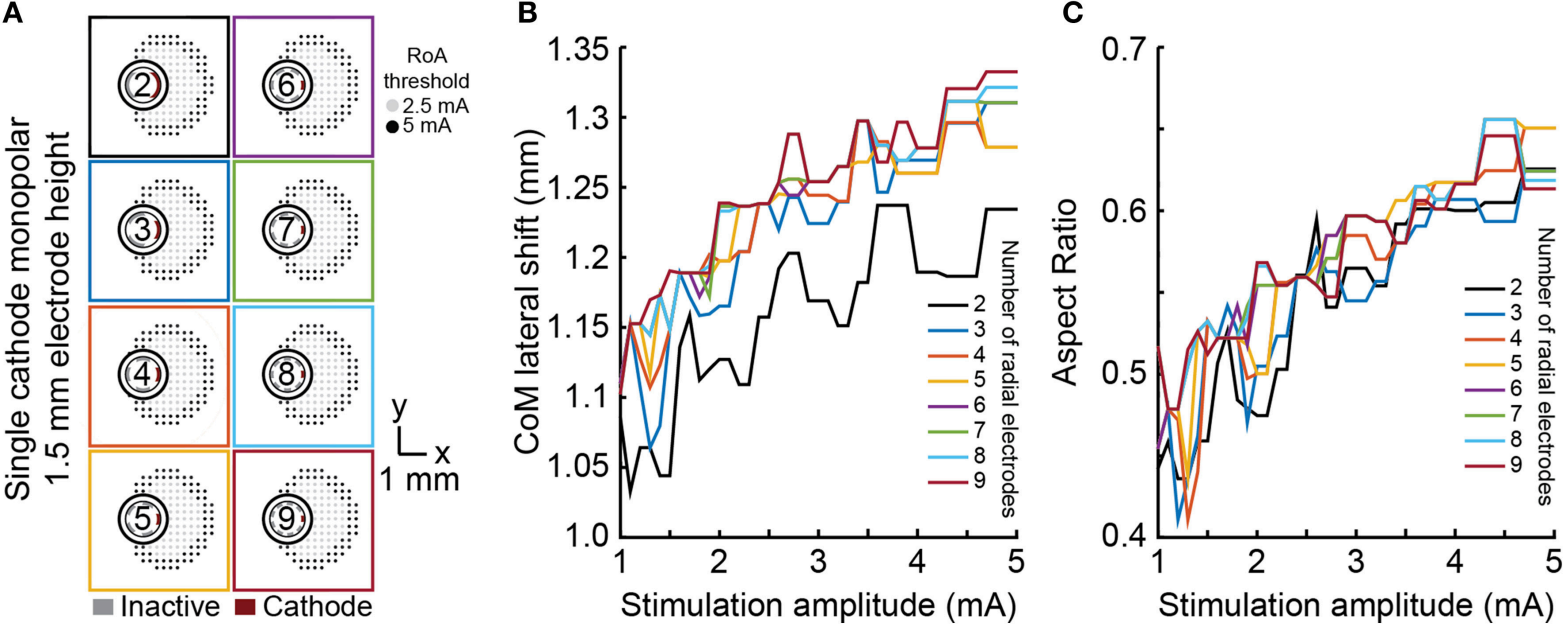
**Monopolar single-cathode lateral shift and aspect ratio**. RoA lateral shift and aspect ratio for monopolar single-cathode stimulation using DBSA lead designs with 1.5 mm electrode height within the range of 1–5 mA. Similar RoAs were produced from all DBSA designs **(A)**. As stimulation amplitude was increased, lateral shift and aspect ratio both increased at similar rates **(B,C)**.

Lateral shift, angular shift, and aspect ratio were used to evaluate the ability of each lead to shift, steer, and sculpt a RoA using monopolar stimulation within the range of 1–5 mA. Lateral shift for cylindrical shell electrodes did not significantly vary from zero as they produced a radially symmetric RoA. For all DBSA lead designs, at 1 mA, lateral shift increased from 0 mm to ~1.1 mm, regardless of the number of radial electrodes (Figure 5) or electrode height. Lateral shift increased moderately from 1.1 mm to 1.3 mm with stimulation amplitude increasing beyond 1 mA for all DBSA lead designs. Aspect ratio increased with stimulation amplitude at a similar rate for DBSA lead designs with the same electrode height (Figure 5). Electrodes with shorter heights were found to produce a slightly more circular RoA resulting in an aspect ratio closer to 1. For instance, the mean aspect ratio at 1 and 5 mA increased from 0.48 and 0.63 for DBSAs with 1.5 mm electrodes to 0.51 and 0.65 for DBSAs with 0.5 mm electrodes.

**Figure 6:**
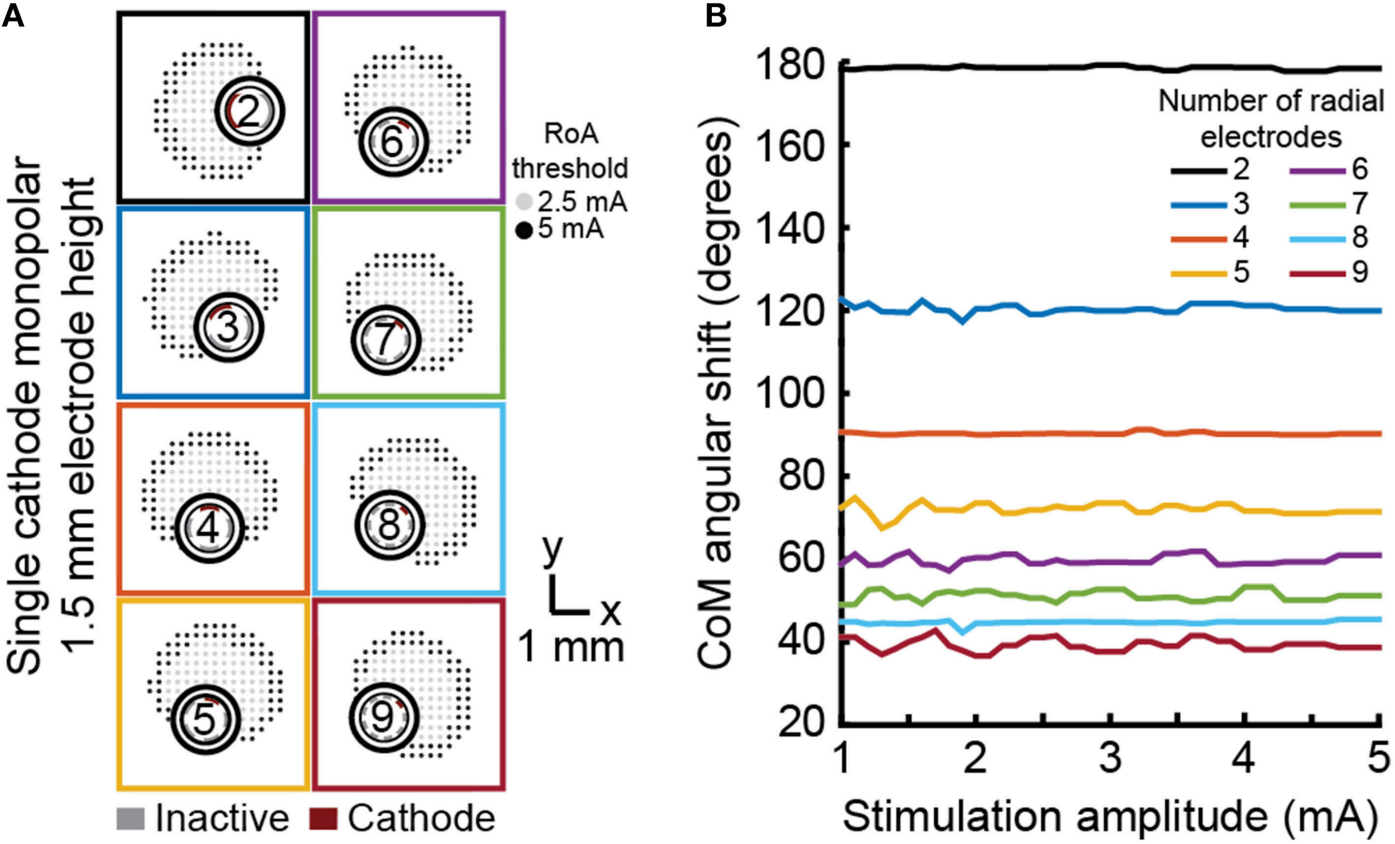
**Monopolar single-cathode steering**. Angular shift for monopolar single-cathode stimulation using DBSA lead designs with 1.5 mm electrode height within the range of 1 to 5 mA. DBSA leads with more electrodes were capable of finer RoA CoM angular shifting **(A)** in accordance with electrode angular separation **(B)**.

Angular shift varied in accordance with angular separation of electrodes (Figure 6). For example, the six radial electrode lead incorporated electrodes separated by 60° and the RoA CoM angular shift resulting from stimulation through neighboring contacts was calculated to be 60°. Angular shift did not vary for leads with different electrode height nor did it vary with stimulation amplitude.

**Figure 7:**
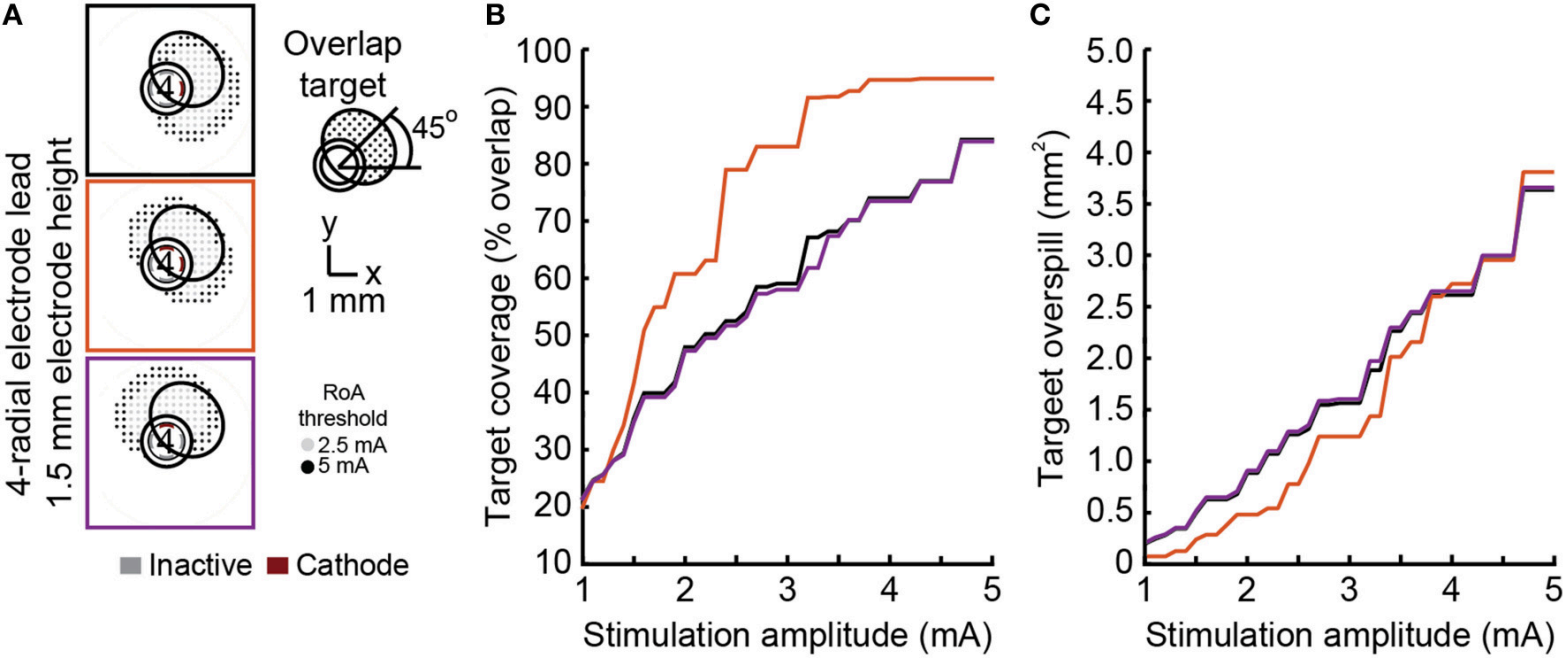
**Steering toward an offset target region**. Steering activation toward a target region between electrodes was investigated using DBSA–e4–h1.5 with single-cathode and multi-cathode stimulation configurations **(A)**. The multi-cathode configuration performed best with a 45° angular shift **(B)** and exhibited the largest overlap and smallest overspill for any given stimulation amplitude **(C)**.

None of the stimulation configurations tested resulted in complete coverage of a rotated target region without moderate to large overspill (Figure 7). The dual cathode configuration performed the best overall. The angular shift for this configuration was closest to 45° and target coverage was highest with the lowest spillover.

### Shifting and sculpting activation with multi-cathode monopolar DBS

**Figure 8:**
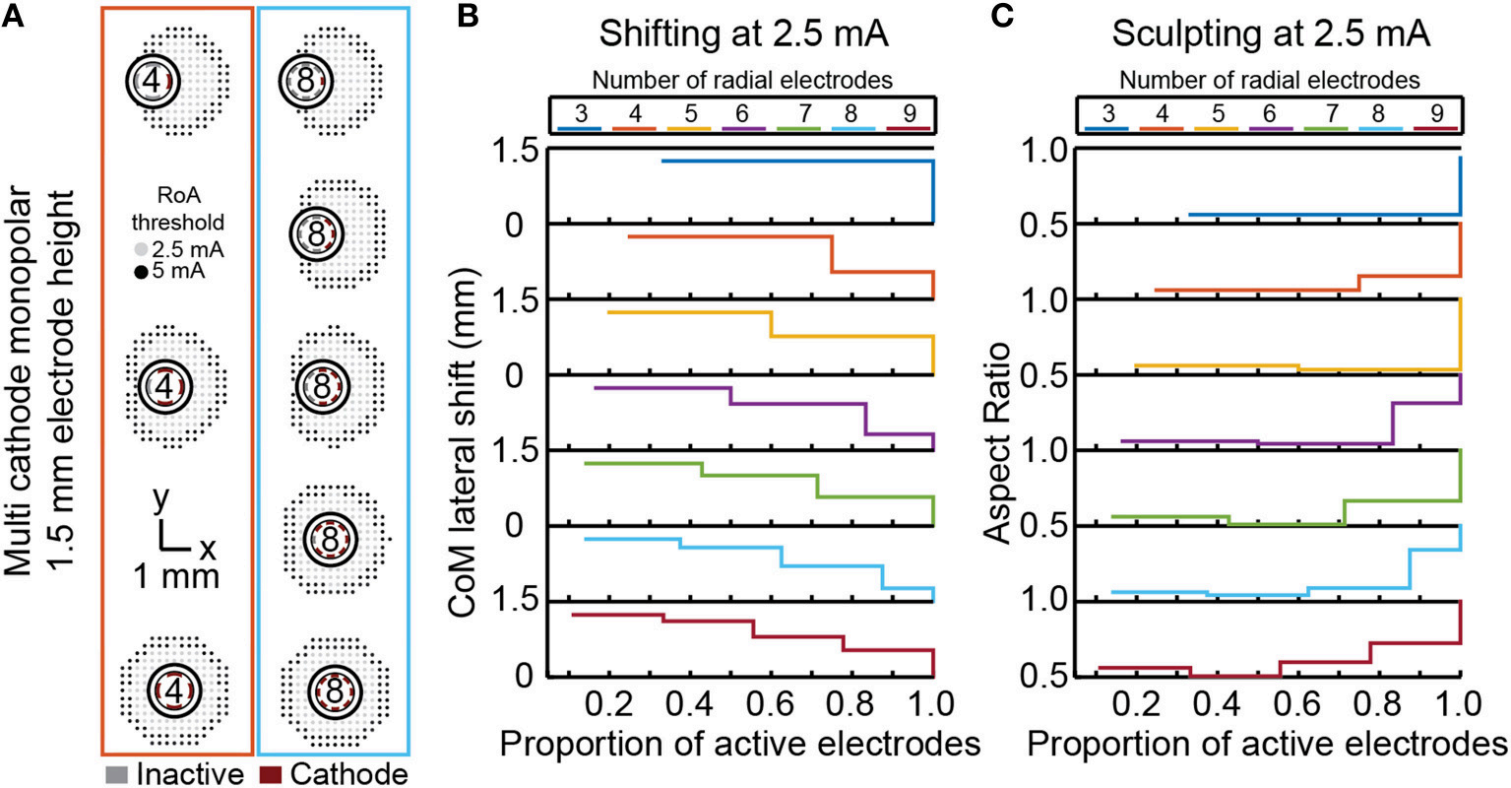
**Incremental CoM shifting using monopolar multi-cathode stimulation**. Monopolar stimulation currents were uniformly split across an increasing number of radial electrodes for each DBSA **(A)**. DBSAs with more radial electrodes enabled shifting within the same range but at improved resolution **(B)**. Aspect ratio decreased initially for DBSAs with more than 4-radial electrodes and increased from ~0.5 to 1 as the proportion of active electrodes increased **(C)**.

For each DBSA lead design, uniform multi-cathode stimulation using a larger proportion of available radial electrodes enabled shifting of the RoA CoM from 0, lead-center, to ~1.3 mm in the direction of the primary cathode. The resolution with which RoA CoM could be shifted from one extreme to the other increased as the number of radial electrodes increased (Figure 8). Lateral shift increased slightly for larger stimulation amplitudes and did not change with electrode height. Increasing the proportion of active electrodes first decreased then increased aspect ratio for leads with more than four radial electrodes. The initial decrease in aspect ratio was a result of added cathodes facing the same direction as the center-most cathode. In general, increasing the proportion of active electrodes increased the aspect ratio toward one, indicating a more radially uniform RoA. These trends were found to be consistent for DBSA lead designs with different electrode heights.

**Figure 9:**
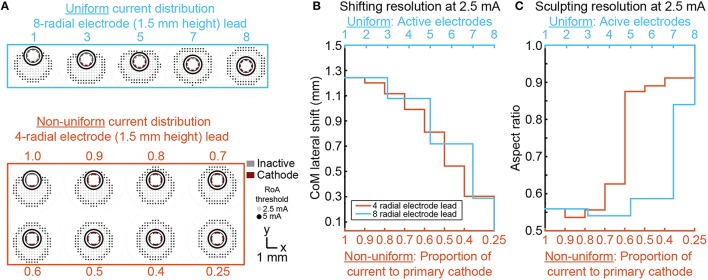
**Multi-cathode, non-uniform current shifting of the CoM**. Monopolar stimulation currents uniformly split across an increasing number of radial electrodes using DBSA–e8–h1.5 compared to monopolar stimulation non-uniformly split across electrodes using DBSA–e4–h1.5 **(A)**. Non-uniform configurations using DBSA–e4–h1.5 resulted in improved shifting resolution in comparison to uniform configurations using DBSA–e8–h1.5 **(B)**. Aspect ratio profile was similar for the two strategies but was shifted for non-uniform current shifting indicating more circular RoAs were generated from non-uniform shifting **(C)**.

Non-uniform multi-cathode stimulation enabled RoA CoM shifting within the same range as uniform current shifting, but with improvement in shifting resolution (Figure 8). Shifting resolution approximately doubled non-uniform multi-cathode stimulation using DBSA–e4–h1.5 in comparison to uniform multi-cathode stimulation using DBSA–e8–h1.5. The aspect ratio range was approximately the same for uniform and non-uniform multi-cathode stimulation; however, the aspect ratio profile shifted to the left indicating that the non-uniform multi-cathode stimulation produced more circular RoAs (Figure 9).

### Classification

**Figure 10:**
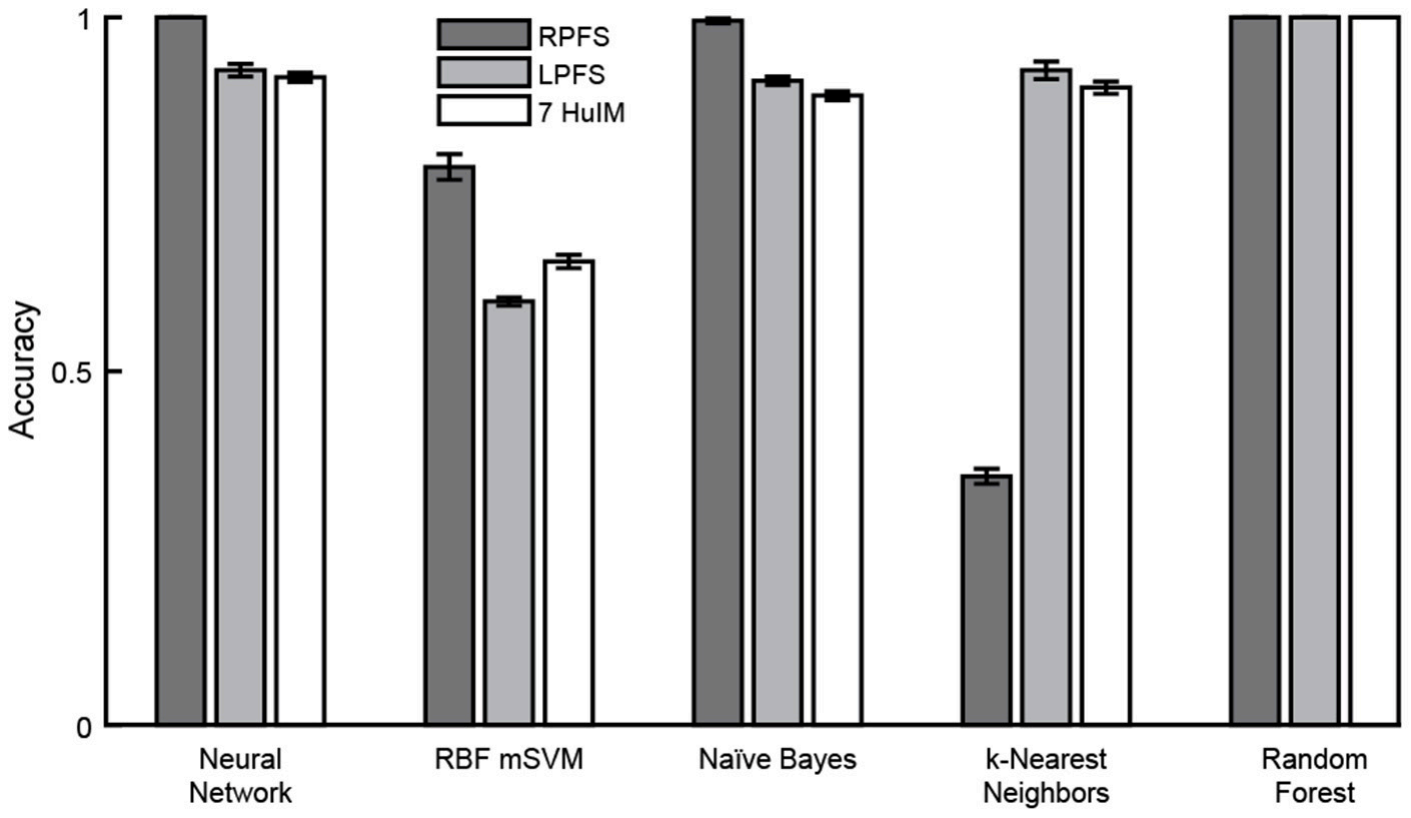
**Classification accuracy**. Mean classification accuracy and accuracy standard error (represented by error bars) were calculated for each classifier/feature set combination across 10-folds. Perfect classification of monopolar stimulation settings was achieved with the random forest classifier using any of the three feature sets. The neural network, naïve Bayes and random forest classifiers achieved perfect or near perfect accuracy using the region properties feature set.

Cross validation using 10 folds was performed using three feature sets in combination with five machine learning algorithms. In general, high mean classification accuracy was achieved with low standard error across the 10 folds. The random forest classification algorithm, which involves automated feature selection, performed best, achieving perfect classification using any of the three feature sets (Figure 10). Of the remaining classifiers where no feature selection/reduction was performed: the neural network classifier achieved perfect accuracy and the naïve Bayes classifier achieved accuracy above 0.95 using the RPFS. Classification using the RPFS produced the highest accuracy for all except in the case of the *k*-nearest neighbors classifier. The LPFS and 7 HuIM feature set achieved similar accuracy when used in combination with the neural network, naïve Bayes and *k*-Nearest neighbors classifiers.

### Feature importance

**Figure 11:**
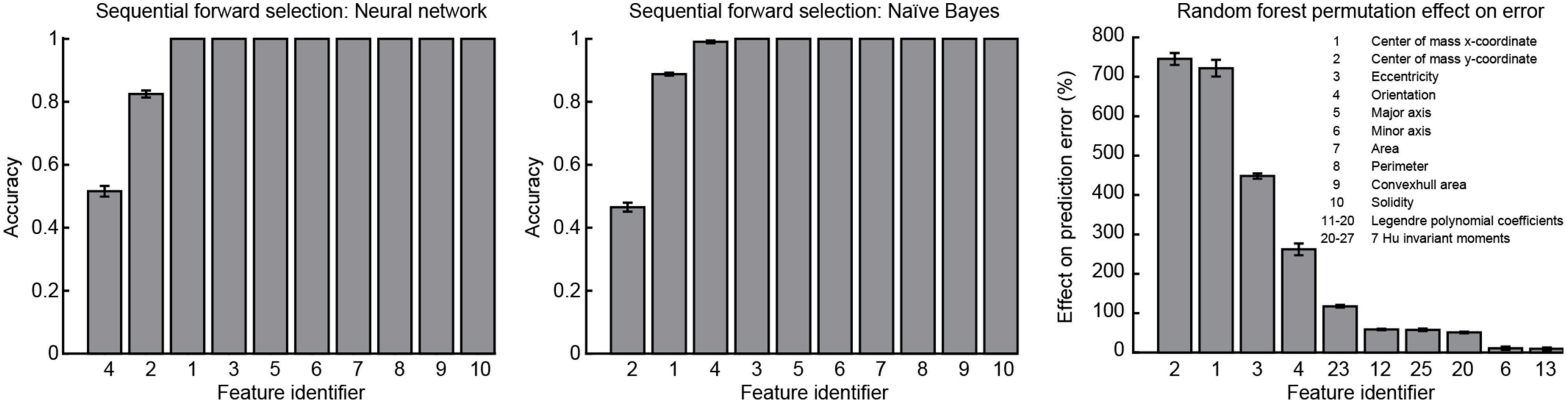
**Feature importance**. Sequential forward selection accuracy converged to one after the addition of features 1, 2, and 4 using both the neural network and naïve Bayes classifiers. From the random forest algorithm, the effect on classification error was increased most by the random permutation of features 1, 2, 3, and 4. Features 1: CoM *x*-coordinate, 2: CoM *y*-coordinate, and 4: ellipse fit orientation were found to be the most important features and using only these three features in combination with the neural network and naïve Bayes classifiers enabled perfect classification.

Sequential forward selection and results from the random forest classification algorithm were used to evaluate feature importance. Mean accuracy was calculated as an indicator of feature importance at each stage of the forward selection for both the neural network and naïve Bayes classifiers. From the random forest algorithm, mean effect on prediction error resulting from random permutation of each feature across the out-of-bag samples was used as an indicator of feature importance. A low standard error was calculated for all indicators of feature importance. Using either the neural network or naïve Bayes classifier, mean accuracy converged to one after the addition of the same four features: CoM *x*-coordinate, CoM *y*-coordinate, ellipse fit eccentricity and ellipse fit orientation. These same four features were ranked as the most important by the random forest algorithm (Figure 11). Although all features were included in the analysis, forward selection using the neural network and naïve Bayes classifiers resulted in the most important features being from only the RPFS.

## Discussion

While DBS therapy is often successful in managing the symptoms of a range of medication-refractory brain disorders, the spatial precision with which the therapy can be delivered using a conventional lead with cylindrical shell electrodes can be limiting for cases of slight neurosurgical targeting error or for brain regions with complex morphologies. Previous studies have developed methodologies to steer and direct activation volumes along a DBS lead studies (Wei and Grill, 2005; Butson and McIntyre, 2008; Chaturvedi et al., 2012; Barbe et al., 2014a,b), but less is known about programming stimulation settings around a DBS lead (Martens et al., 2011). The results of this study show for a DBS lead embedded within or near a fiber tract that: (1) four ellipsoidal electrodes around a DBS lead provided good flexibility to steer, sculpt, and shift a region of neural activation without exceeding the charge storage capacity of platinum-iridium electrodes or charge injection limits for neural tissue, and (2) a small feature set, including only three geometric features representing a target region enabled robust machine learning classification of electrode stimulation configuration.

### DBS array design considerations

Microfabrication processes enable new opportunities to develop stimulating probe technology with many more electrode sites than what is currently in clinical use for DBS applications (Martens et al., 2011; Willsie and Dorval, 2015; Connolly et al., 2016). Increasing the number of electrodes and in turn decreasing the size of electrodes has several important effects on the region of neural tissue including limiting the spatial extent of the RoA due to charge storage capacity and charge injection limits (Merrill et al., 2005; Wei and Grill, 2005). Previous preclinical studies in animal models of neurological disorders have also noted that DBS therapy is partially based on modulating the neuronal firing patterns of a fairly large volume of tissue (Johnson and McIntyre, 2008; Johnson et al., 2012) within a target volume (Butson et al., 2007). Thus, while increasing the number of electrodes may provide more spatially focused stimulation, generating a therapeutic effect through DBS arrays is likely to require grouping electrodes together for high-density DBS arrays. This grouping approach would be complicated by radial diffusion properties that result in higher charge densities near the edges of each electrode in a group (Wei and Grill, 2005; Howell and Grill, 2014).

In this study, we extend these results showing that charge storage capacity and charge injection are limiting factors, though to different extents as the number of radially electrodes is increased. For DBSA designs with small electrode surface areas, advanced electrode coatings (Cogan et al., 2004; Cogan, 2008; Luo et al., 2011) may address the issue of charge storage capacity, but the charge injection limits will remain an issue as was shown for DBSA lead designs with five or more radial electrodes. Elliptical electrodes with height ranging from 0.5 to 1.5 mm and width ranging from 0.2 to 0.9 mm were used in this study. For electrodes with the largest height and the smallest width, it is possible that these highly eccentric electrodes would have higher charge density values at the ends of the electrode major axis (Grill and Wei, 2009) similar to how large current density values are found at the corners of rectangular electrodes (Wei and Grill, 2005).

### Shaping the region of activation

One of the primary motivations for advances in DBS lead and stimulator designs is to enable compensation for sub-optimally placed leads. Ideally, leads with cylindrical shell electrodes are implanted with one of the electrodes at the geometric center of the neural target enabling good stimulation coverage with minimal overspill. With targets that are several centimeters deep and only millimeters across, precise lead placement can be challenging. With a cylindrical shell electrode, a small offset in the final lead location may significantly limit stimulation efficacy and result in stimulation induced side effects resulting from activation of nearby pathways. DBSAs have been proposed as able to compensate for such placement issues (Martens et al., 2011; Contarino et al., 2014; Pollo et al., 2014). As we have shown, monopolar stimulation through a single radial electrode resulted in a 1–1.3 mm RoA CoM lateral shift and increasing stimulation amplitude minimally affected the CoM location. Additional radial electrodes or proportional current steering provided options to incrementally shift the RoA CoM with sub-millimeter resolution, but in cases where more than a 1 mm shift in the RoA CoM is needed for compensation of lead misplacement, this need would not be adequately addressed by any of the DBSA designs evaluated in this study. The results showed that uniform multi-cathode stimulation enabled incremental CoM shifting, but was limited by the number of available radial electrodes. Non-uniform multi-cathode stimulation resulted in better shifting resolution with four radial electrodes than could be achieved using uniform multi-cathode stimulation with eight radial electrodes. From this we conclude that fewer electrodes does not limit shifting if non-uniform stimulation strategies are used. However, practical implementation of non-uniform stimulation requires fine and independent control of multiple stimulation channels. In regard to lead design, leads with fewer radial electrodes may be preferable because of larger electrode surface areas and possibly less complex manufacturing processes. In regard to implantable pulse generator design, fewer independent current sources may be preferable to allow for device miniaturization.

Stimulators with independent current-regulated channels are well-established in the fields of spinal cord stimulation for pain mediation (Hegarty, 2011), auditory nerve stimulation for hearing restoration (Wilson and Dorman, 2008), and retinal stimulation for vision restoration (Matteucci et al., 2013). The advent of stimulators with independent channels in these fields have prompted significant research into the utility of various stimulation strategies for directing and focusing current, particularly in the case of auditory nerve stimulation, where highly conductive fluid separates the stimulating electrodes from the stimulation target (Dallas, 1992). Strategies for steering and focusing stimulation include the use of multiple sources to steer a region of neural activation and the use of bipolar stimulation to narrowly focus current (Berenstein et al., 2008; Bonham and Litvak, 2008). These strategies have been implemented with varying degrees of success for cochlear implants and spinal cord stimulation. These strategies have also been investigated in DBS systems via modeling studies (Butson and McIntyre, 2008; Chaturvedi et al., 2012) and clinical studies (Barbe et al., 2014a,b) for the purpose of steering neural activation along the length of a conventional DBS lead. Our results indicate that for steering, shifting, and sculpting of neural activation around the lead, a DBSA with four electrodes per row combined with a pulse generator that has independent current sources for each electrode would be highly effective at steering and shifting a region of neural activation around a DBSA lead. Our results also indicate that more than four electrodes would be minimally advantageous.

Radial shifting and steering have potential to benefit clinical outcomes for a number of DBS targets (Montgomery, 2010). For instance, the subthalamic nucleus target for Parkinson's disease is adjacent to the corticospinal tract of internal capsule (Chaturvedi et al., 2012) and non-motor territories of the subthalamic nucleus (Frankemolle et al., 2010) that when stimulated can lead to adverse side effects. The ventral intermediate nucleus of thalamus, which is the primary target for treating Essential Tremor, is adjacent to the internal capsule, the somatosensory nucleus of thalamus, and non-motor pathways involved in language and cognition (Herrero et al., 2002). Similarly, the pedunculopontine tegmental area is replete with adjacent fibers of passage including the superior cerebellar peduncle, medial and lateral lemnisci, and the central tegmental tract among others that may have confounding effects on treatment of medication-refractory gait disorders (Zitella et al., 2013). Radial current shifting and steering may also have important applications to DBS targets that are embedded within fiber tracts including those for depression (Riva-Posse et al., 2014), obsessive compulsive disorder (Greenberg et al., 2006), and memory disorders (Hamani et al., 2008).

### Machine learning to facilitate programming

Along with greater flexibility in directing neural activation, DBS arrays present exponentially more options during programming. This necessitates the use of (1) guided programming through computational algorithms (Chaturvedi et al., 2013; Xiao et al., 2016), and (2) empirical algorithms that rely on the spatial distribution of electrophysiological biomarkers (Little and Brown, 2012). Here, we investigated feature sets to be used in building machine learning classifiers for predicting DBSA stimulation settings. These feature sets were constructed from the two-dimensional computational modeling results of axonal activation using the DBSA–e4–h1.5 lead and relied upon computer vision feature extraction techniques. In computer vision, feature extraction is commonly performed to identify objects that may be “viewed” by a machine using images or video that was captured and processed internally. Robust computer vision identification requires that objects be identifiable when viewed at different distances, angles, and locations within the field of view requiring the use of scale, rotation, and translation invariant feature sets (Lowe, 1999). The feature sets we have designed for use in machine learning classifiers for DBS rely on these same principles, but include a center of mass estimate that is relative to the lead-center so that changes in the RoA direction and shift may be detected. In addition to investigating the value of various features for such classification algorithms, we have demonstrated robust machine learning classification of electrode stimulation configuration using a single row of electrodes. Our investigation into feature sets revealed that excellent classification could be achieved using a small number of two dimensional geometric features that may be readily translated in three-dimensional geometric measures. Running axon model simulations, feature extraction, and classifier training required significant computation time, but the resulting five classification algorithms were able to be deployed in less than 1 min using a conventional desktop computer. The speed with which such algorithms can be deployed demonstrates the power and practicality of such algorithms for use in clinical DBS programming.

### Limitations

The quasistatic finite element models used for predicting tissue voltage in this study were idealized as isotropic and were homogeneous within bulk neural tissue. Increasingly complex models that more precisely model tissue conductivity using diffusion weighted imaging have been introduced in the past decade and have been shown to impact biophysical simulation results (Butson et al., 2007; Chaturvedi et al., 2010; Zitella et al., 2015), particularly for modeling of electrical stimulation near white matter fiber tracts (Butson et al., 2007; Schmidt and van Rienen, 2012). Further, the conductance values utilized in the tissue models presented here rely on experimentally determined values for conductance that are subject to uncertainty as evident by the range of values reported within the scientific literature (Gabriel C. et al., 1996; Faes et al., 1999). Variations of tissue conductance within the range of reported values have been shown to lead to significant uncertainty in the activation predictions of biophysical models (Schmidt et al., 2013). Additionally, stimulus waveforms propagating through encapsulation and brain tissue are likely to be influenced reactive tissue impedances (Johnson et al., 2005; Otto et al., 2006; Williams et al., 2007; Yousif and Liu, 2009) and the quasistatic model does not incorporate this feature. Using the modeling framework presented here, future work may assess the impact of variations in conductance, brain anisotropy, and reactive tissue response on the DBSA design and feature selection for model based programing algorithms.

The multi-compartment axon models used in this study were idealized straight cables coursing parallel to the DBS lead. Modeling work with straight axons has potential utility for DBS targets that are within or near large fiber tracts that have minimal curvature (Greenberg et al., 2006; Hamani et al., 2008; Blomstedt et al., 2010; Riva-Posse et al., 2014). However, it is important to consider that this idealized model geometry lacks the anatomical trajectories known to occur in many targets of DBS. In these cases, factors such as stimulating regions with networks of cellular and axonal processes (Zitella et al., 2013), inducing complex cellular entrainment patterns (Hashimoto et al., 2003; Agnesi et al., 2013), and increasing the likelihood of axonal conduction failure due to axonal branching (Debanne, 2004), lack of myelination (Chomiak and Hu, 2007), and synaptic fatigue (Rosenbaum et al., 2014) should be considered.

Elimination of the ETI from the finite element models relied on a subset of simulations that incorporated an ETI equivalent circuit model that assumed the electrode material was platinum-iridium. To avoid exceeding the charge storage capacity of the electrodes with a clinically acceptable factor of safety, realistic lead designs with small electrodes would likely require the use of coatings such as iridium oxide (Cogan, 2008), PEDOT (Ludwig et al., 2011), or TiN (Weiland et al., 2002) for which lumped ETI equivalent circuit model values would likely differ.

## Conclusions

DBS arrays with radially distributed electrodes have potential to improve patient outcomes by enhancing the flexibility of directing stimulation around an implanted DBS lead. Clinical DBS leads with cylindrical shell electrodes do not exceed electrode charge storage capacity or charge injection limits due to the large surface area and existing voltage or current compliances of current implantable pulse generators. However, segmenting the cylindrical shell electrode design into two or more electrodes around the lead circumference would bring these stimulation limits into consideration. For DBSAs, monopolar single-cathode stimulation was useful for shifting the RoA CoM from lead-center to 1.3 mm in the direction of the stimulating electrode. Shifting resolution on the scale of 0.1 mm was achievable with four radial electrodes using non-uniform distribution of current, suggesting a higher density DBSAs would not be needed to achieve clinically relevant RoA shifting if independent current sources are utilized. A simple feature set consisting of the RoA center of mass and orientation enabled robust machine learning classification with accuracy equal to 1 for a range of monopolar stimulation settings.

## Author contributions

Designed model framework: BT, LZ, YX, MJ. Performed model simulations: BT, LZ, YX. Analyzed the data: BT, MJ. Wrote the manuscript: BT, MJ.

## Funding

This study was supported by the Michael J Fox Foundation, the University of Minnesota's MnDRIVE (Minnesota's Discovery, Research and Innovation Economy) initiative, NIH R01-NS081118, NSF-IGERT (Systems Neuroengineering, DGE-1069104), and NSF-GRFP (00006595 to BT).

### Conflict of interest statement

The authors declare that the research was conducted in the absence of any commercial or financial relationships that could be construed as a potential conflict of interest.
